# The role of AI capabilities and entrepreneurial competency in SME performance: the mediating role of strategic intelligence

**DOI:** 10.3389/frai.2026.1833033

**Published:** 2026-07-13

**Authors:** Shrooq Alsenan, Waleed Mugahed Al-Rahmi, Ibrahim Youssef Alyoussef, Nisar Ahmed Dahri

**Affiliations:** 1 Information Systems Department, College of Computer and Information Sciences, Princess Nourah bint Abdulrahman University, Riyadh, Saudi Arabia; 2 Department of Management Information System, College of Business Administration, Dar Al Uloom University, Riyadh, Al Falah, Saudi Arabia; 3 Education Technology, Faculty of Education, King Faisal University, Al-Ahsa, Saudi Arabia; 4 Faculty of Educational Sciences and Technology, Universiti Teknologi Malaysia, Skudai, Johor, Malaysia; 5 Sohar University, Sohar, Oman

**Keywords:** artificial intelligence capabilities, entrepreneurial competency, PLS-SEM, SME performance, strategic intelligence

## Abstract

**Introduction:**

This study examines the effects of Artificial Intelligence (AI) capabilities and entrepreneurial competency on Small and Medium Enterprise (SME) performance through the mediating role of strategic intelligence in SMEs operating in Malaysia and Saudi Arabia. Grounded in Resource Orchestration Theory and Core Competency Development Theory, the study explores how AI integration skills, smart decision-making abilities, innovativeness, and risk management competencies contribute to enhanced organizational performance in an increasingly digital business environment.

**Methods:**

A cross-sectional quantitative research design was adopted. Data were collected from 320 SMEs in Malaysia and Saudi Arabia using a structured questionnaire. The measurement model and structural relationships were analyzed using Partial Least Squares Structural Equation Modeling (PLS-SEM). The study examined direct relationships between AI capabilities, entrepreneurial competency, strategic intelligence, and SME performance, as well as the mediating effect of strategic intelligence.

**Results:**

The findings reveal that both AI capabilities and entrepreneurial competency have a significant positive impact on strategic intelligence and SME performance. Furthermore, strategic intelligence was found to significantly mediate the relationship between AI capabilities, entrepreneurial competency, and SME performance. These results indicate that SMEs with stronger AI-driven capabilities and entrepreneurial competencies are more likely to develop higher strategic intelligence, which in turn enhances overall organizational performance.

**Discussion:**

The study contributes to the growing body of literature on AI-driven organizational capabilities by providing empirical evidence from developing economies, specifically Malaysia and Saudi Arabia. The results highlight the importance of integrating AI competencies with entrepreneurial skill development to improve strategic decision-making and business competitiveness. Practically, the findings suggest that SME managers and policymakers should prioritize AI adoption and entrepreneurial capability development as key strategies for improving innovation, resilience, and long-term performance in SMEs.

## 1 Introduction

In the current dynamic and technology-driven business environment, organizations increasingly rely on artificial intelligence (AI) to improve operational efficiency, decision-making quality, and innovation capability. AI technologies such as machine learning, predictive analytics, natural language processing, and intelligent automation are transforming how firms manage resources, respond to market uncertainty, and sustain competitiveness. Recent studies indicate that AI adoption can enhance organizational agility, knowledge sharing, and strategic responsiveness, particularly in digitally transforming business environments (Mikalef et al., 2023; Soomro et al., 2024). However, the benefits of AI adoption are not automatically realized, especially among small and medium-sized enterprises (SMEs), which often face resource limitations, technological barriers, and managerial capability constraints.

SMEs are widely recognized as major contributors to economic development, employment generation, and innovation across both developed and developing economies. In Malaysia and Saudi Arabia, SMEs play an essential role in supporting economic diversification and long-term national development strategies. Malaysia has strengthened its digital economy agenda through entrepreneurship and innovation initiatives (Ismail et al., 2022), while Saudi Arabia’s Vision 2030 emphasizes digital transformation and SME empowerment as key drivers of economic sustainability and competitiveness (Abdelwaheed et al., 2025). Despite these policy initiatives, SMEs in both countries continue to face challenges related to digital readiness, technological expertise, and strategic resource allocation. These challenges increase the need for SMEs to develop AI capabilities alongside entrepreneurial competencies that can support adaptation and sustainable growth in uncertain environments.

Recent literature suggests that organizational readiness, technological preparedness, leadership support, and digital capabilities are critical determinants of successful AI adoption in SMEs. Studies from Malaysia indicate that AI adoption is strongly influenced by technological readiness, financial preparedness, and digital literacy (Chuan-Fu and Kuppusamy, 2025; Keong et al., 2025), while SMEs in Saudi Arabia place greater emphasis on leadership support, government facilitation, and strategic agility (Badghish and Soomro, 2024). Although AI technologies provide SMEs with opportunities to improve operational efficiency and customer engagement, the implementation process remains difficult due to limited expertise, financial constraints, resistance to change, and insufficient managerial capabilities (Ahmad et al., 2022). These barriers are particularly significant for SMEs because they generally lack the financial and technological resources available to larger firms.

AI capabilities are increasingly viewed as strategic organizational resources that enhance decision-making and business intelligence. Through predictive analytics and data-driven systems, SMEs can improve customer relationship management (Mahmud et al., 2025), supply chain coordination, market forecasting, and operational planning. AI-enabled technologies also support real-time analysis of customer preferences and competitive trends, allowing firms to respond more effectively to market changes (Basri, 2020). However, technological capability alone may not guarantee superior organizational performance. Existing studies increasingly argue that entrepreneurial competency plays a critical role in enabling firms to effectively leverage digital technologies and transform technological investments into strategic outcomes.

Entrepreneurial competency refers to the ability of organizational leaders and entrepreneurs to identify opportunities, manage risks, innovate, and adapt to changing business conditions. Entrepreneurial competencies include strategic thinking, innovativeness, decision-making ability, resilience, and opportunity recognition (Ismail et al., 2022). These competencies are particularly important in SMEs because owner-managers often play central roles in organizational strategy and technology adoption decisions. Recent studies from Malaysia and Saudi Arabia suggest that entrepreneurial orientation, managerial competence, and digital literacy significantly influence SME innovation and sustainability outcomes (Mohamad et al., 2025; Seraj et al., 2022). In addition, entrepreneurial competency strengthens firms’ ability to manage uncertainty and respond proactively to technological disruptions.

The convergence of artificial intelligence capabilities and entrepreneurial skills could enhance strategic intelligence in SMEs. Strategic intelligence involves an organization’s ability to gather, analyze, and implement strategic information in its decision-making processes. This allows the firm to detect changes in its environment, predict risks, and make informed strategic decisions. The use of artificial intelligence helps in the process by providing valuable information on market dynamics and future events, while entrepreneurial skills allow managers to apply this information in their decision-making. In essence, strategic intelligence could be considered an intermediary variable between technological capacity and organizational success.

Strategic intelligence has become increasingly important for SMEs in Malaysia and Saudi Arabia owing to the rapidly changing business and competitive environment due to the adoption of digital transformation policies (Haq and Suki, 2025; Mahmud et al., 2025). This requires SMEs to develop not only technological but also strategic intelligence skills. Research shows that knowledge management practices, leadership endorsement, and organizational readiness facilitate the positive connection between the use of AI and innovation success. Likewise, strategic intelligence has been positively associated with innovation capacity and organizational agility among SMEs (Tan et al., 2025).

Although research on AI adoption and SME digital transformation has grown substantially in recent years, several important gaps remain in the literature. First, most previous studies focus primarily on large organizations, while the unique challenges faced by SMEs in developing economies remain underexplored. SMEs often operate under resource constraints and require flexible and low-cost technological solutions that differ significantly from large enterprises. Second, existing studies generally examine AI adoption, entrepreneurial competency, or organizational performance independently rather than integrating these constructs within a unified framework (Abdelwahed and Alshaikhmubarak, 2023; Ismail et al., 2022). Third, the mediating role of strategic intelligence between AI capabilities, entrepreneurial competency, and SME performance has received limited empirical attention, particularly in the contexts of Malaysia and Saudi Arabia.

This study bridges the knowledge gaps identified by conducting an analysis of the relationships between the capabilities of AI, entrepreneurial competency, strategic intelligence, and SME performance based on the theoretical framework of Resource Orchestration Theory (ROT). According to this theory, organizations can achieve competitive advantage and effectiveness by integrating, coordinating, and harnessing strategic resources. As explained by Yang et al. (2025), it is important for SMEs to not only obtain resources related to technology but also orchestrate their capabilities related to management, personnel, and strategy in order to be successful in their efforts at digital transformation. Recent empirical research suggests that when AI capabilities are complemented by entrepreneurial orientation and organizational readiness, SMEs become more innovative, sustainable, and resilient (Alshuaibi et al., 2024; Yang et al., 2025).

This study makes significant contributions to the field of research in a number of ways. Firstly, it constructs an integrated model explaining how AI capabilities, entrepreneurial competency, strategic intelligence, and SME performance are related to each other within the frameworks of the SME environments in Malaysia and Saudi Arabia. Secondly, it applies and expands Resource Orchestration Theory by considering how SMEs can strategically combine technological resources with entrepreneurial resources to enhance their performance. Thirdly, the mediating effect of strategic intelligence in the relationship between AI capabilities and business performance is empirically tested in this study.

Accordingly, this study aims to examine the effects of AI capabilities and entrepreneurial competency on strategic intelligence and SME performance. In addition, the study investigates the mediating role of strategic intelligence within these relationships. The findings are expected to provide both theoretical and practical insights into how SMEs can leverage AI technologies and entrepreneurial capabilities to improve long-term competitiveness and organizational sustainability.

## 2 Theoretical model and hypothesis development

This study is grounded in Resource Orchestration Theory (ROT) and Core Competency Development Theory to explain how AI capabilities, entrepreneurial competency, and strategic intelligence contribute to SME performance in Malaysia and Saudi Arabia. ROT suggests that organizational success depends not only on possessing valuable resources but also on effectively structuring, bundling, and leveraging those resources to create competitive advantage (Sirmon et al., 2011). This perspective is highly relevant for SMEs because firms in developing economies often operate under financial, technological, and managerial constraints. Therefore, SMEs must strategically coordinate technological and human resources to remain competitive in increasingly digital business environments. Recent studies confirm that SMEs in Malaysia and Saudi Arabia rely heavily on leadership support, digital readiness, and strategic resource allocation to improve innovation and sustainability outcomes.

ROT provides a process-oriented explanation of how organizations integrate and deploy strategic resources to improve adaptability and innovation performance (Alshuaibi et al., 2024). Compared with Dynamic Capability Theory (DCT), which broadly emphasizes sensing and responding to environmental change, ROT offers deeper insight into how firms operationalize and coordinate resources in practice. Similarly, technology adoption theories such as the Technology Acceptance Model (TAM), Unified Theory of Acceptance and Use of Technology (UTAUT), and Technology–Organization–Environment (TOE) framework mainly focus on technology acceptance and adoption factors, including perceived usefulness, compatibility, and organizational readiness. However, these frameworks provide limited explanation regarding how firms strategically combine technological and managerial capabilities to achieve sustainable performance. Recent studies show that organizational readiness, entrepreneurial orientation, and strategic decision-making play a stronger role than environmental factors in AI adoption among SMEs in Malaysia and Saudi Arabia (Ahmed et al., 2025; Alshaibani et al., 2025).

In addition to ROT, Core Competency Development Theory provides a useful explanation for understanding AI capabilities and entrepreneurial competency as strategic organizational competencies. According to this theory, firms gain sustainable advantage through integrated technological expertise, managerial knowledge, and organizational learning capabilities (Javalgi and Todd, 2011). AI capabilities, such as predictive analytics, intelligent automation, and AI-driven decision systems, improve operational efficiency and strategic responsiveness. Recent evidence suggests that AI-enabled digital transformation improves innovation, customer engagement, and operational performance among SMEs when firms possess sufficient technological readiness and leadership support (Aljabari et al., 2024).

Entrepreneurial competency represents another critical strategic capability. It includes innovativeness, risk management, opportunity recognition, and strategic decision-making skills that enable SMEs to adapt to dynamic business conditions. Previous studies in Malaysia and Saudi Arabia indicate that entrepreneurial orientation significantly improves SME innovation and business performance, particularly when supported by absorptive capacity, managerial competency, and organizational flexibility (Ismail et al., 2022). Entrepreneurial competencies also help firms utilize technological resources more effectively, thereby improving resilience and long-term sustainability.

The integration of AI capabilities and entrepreneurial competency may further strengthen strategic intelligence within SMEs. Strategic intelligence refers to the organizational ability to gather, analyze, and utilize strategic information for long-term planning and competitive positioning. AI technologies provide firms with real-time data analytics and forecasting capabilities, while entrepreneurial competency enables managers to convert these insights into strategic actions. Recent studies show that strategic decision-making, absorptive capacity, and entrepreneurial agility mediate the relationship between technological capabilities and SME performance in developing economies (Mahmud et al., 2025; Soomro et al., 2024). Strategic intelligence therefore functions as an important mechanism through which technological and entrepreneurial resources contribute to organizational success.

**Figure 1:**
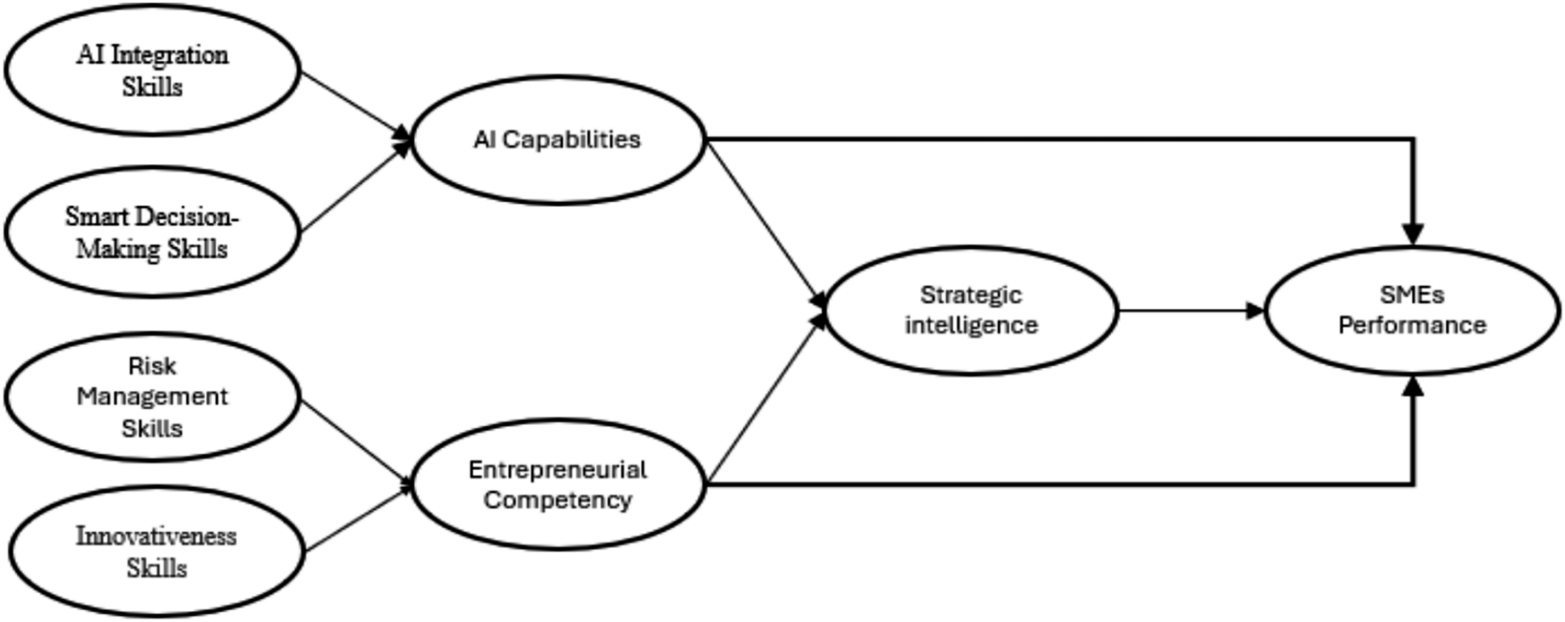
Proposed research model.

This study positions AI capabilities and entrepreneurial competency as strategic enablers of strategic intelligence and SME performance. AI integration skills and smart decision-making capabilities support operational efficiency and digital adaptability, while innovativeness and risk management capabilities strengthen entrepreneurial competency. Strategic intelligence subsequently enables SMEs to align organizational resources with market opportunities and environmental uncertainty. By integrating ROT and Core Competency Development Theory, this study provides a comprehensive framework for understanding how technological and human capabilities jointly contribute to SME sustainability and performance in Malaysia and Saudi Arabia. The study also addresses recent theoretical gaps identified in Scopus-indexed literature regarding the limited integration of AI capabilities, entrepreneurial orientation, and strategic intelligence within SME research models (Figure 1).

### 2.1 Impact of AI integration skills and smart decision-making skills on AI capabilities

AI capabilities refer to an organization’s ability to acquire, integrate, and utilize AI technologies to improve operational efficiency, strategic responsiveness, and organizational adaptability (Aldossari et al., 2025; Keong et al., 2025). For SMEs operating in developing economies such as Malaysia and Saudi Arabia, AI capabilities have become increasingly important because firms face growing pressure to digitalize operations while managing financial, technological, and managerial constraints. The successful development of AI capabilities depends not only on technological investment but also on firms’ ability to effectively integrate AI systems and utilize AI-generated insights in decision-making processes.

Integration capabilities denote a set of abilities of organizations to integrate AI technologies into their business processes and operations. The described capabilities can help in automating processes, data processing, increasing scalability, and making decisions based on factual evidence. According to ROT, competitive advantages do not lie in companies’ possession of technological capabilities but in their efficient coordination and use (Sirmon et al., 2011). Hence, AI technologies will bring benefits to SMEs if they have enough integration capabilities to operationalize their potential in various organizational activities. Earlier research has shown that integration skills such as organizational readiness, employee digital competence, and managerial support are vital in the successful application of AI technologies (Yen et al., 2024).

Another skill that will allow increasing AI capabilities is that of smart decision making. It presupposes the ability to analyze information and make decisions based on its analysis performed with the help of AI-enabled technologies. With the help of AI technologies, companies can get predictive analytics, forecasting, and other useful features. Therefore, making decisions based on such data will become more efficient and beneficial for companies. Moreover, due to limitations in resources, it will be even more relevant to develop smart decision-making capabilities in SMEs.

Core Competency Development Theory further suggests that firms gain sustainable advantage through distinctive organizational capabilities that improve strategic effectiveness. In this regard, smart decision-making skills enhance firms’ ability to strategically utilize AI technologies for forecasting, resource optimization, and opportunity identification (Gangwar et al., 2023). Similarly, Bokhari and Myeong (2022) argued that intelligent decision-making systems strengthen organizational innovation and strategic responsiveness within digitally transforming environments.

**Figure 2:**
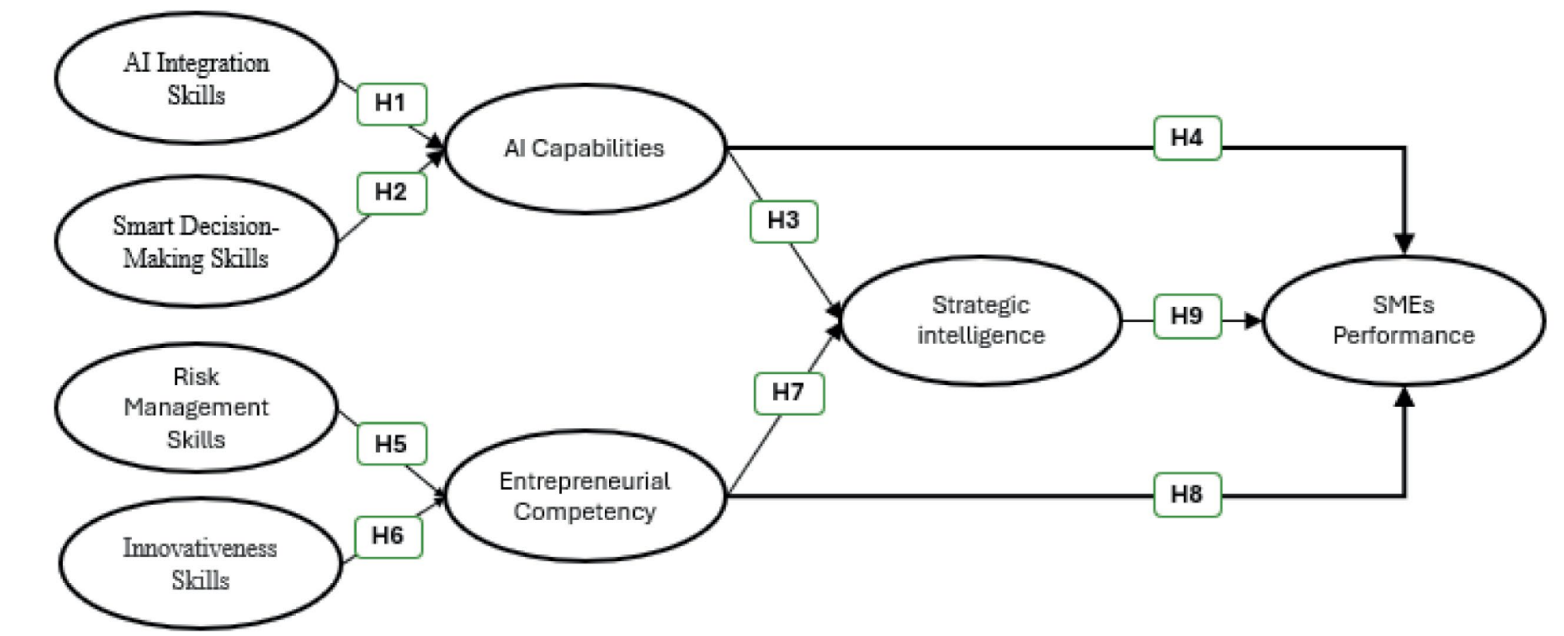
Proposed research hypothesis.

In Malaysia and Saudi Arabia, government-led digital transformation initiatives have encouraged SMEs to strengthen AI adoption and technological readiness (Ahmad et al., 2022; Alattas, 2023). However, the effectiveness of AI implementation continues to depend on firms’ integration capability and managerial competence in utilizing AI-generated insights. SMEs with stronger AI integration and smart decision-making skills are therefore more likely to develop superior AI capabilities and improve organizational adaptability. Accordingly, this study proposes the following hypotheses (Figure 2):

*H1*: AI Integration Skills positively impact AI Capabilities.

*H2*: Smart Decision-Making Skills positively impact AI Capabilities.

### 2.2 Effect of AI capabilities on strategic intelligence, and SME performance

AI capabilities refer to an organization’s ability to acquire, integrate, and utilize AI technologies to support strategic and operational activities (Lada et al., 2023). In SMEs, AI capabilities have become increasingly important for improving organizational adaptability, innovation, and long-term competitiveness. AI-enabled systems support real-time analytics, predictive forecasting, and intelligent automation, allowing firms to process large amounts of information and respond more effectively to changing market conditions. Recent studies indicate that SMEs using AI technologies can improve operational efficiency, customer engagement, and strategic responsiveness (Belhadi et al., 2024).

Strategic intelligence reflects an organization’s ability to gather, interpret, and apply strategic information for long-term planning and decision-making. AI technologies contribute to strategic intelligence by enabling firms to generate timely insights regarding customer behavior, market trends, and competitive risks. This capability is particularly important for SMEs in Malaysia and Saudi Arabia because resource limitations often restrict their ability to conduct large-scale market analysis and strategic forecasting. AI capabilities therefore strengthen SMEs’ ability to improve strategic planning and respond proactively to environmental uncertainty. Recent advances in management, accounting, and economics have emphasized the importance of integrating interdisciplinary approaches to address emerging organizational and economic challenges (Hafinaz and Kumar, 2025).

According to Resource Orchestration Theory (ROT), organizations can attain a competitive advantage not just through resource ownership but also through effective coordination and utilization of the resources (Sirmon et al., 2011). AI capabilities constitute strategic technological resources, which augment the organizational intelligence and adaptability. Therefore, small businesses that can leverage their AI capabilities for strategic purposes are bound to gain an upper hand through enhanced decision-making, market responsiveness, and organizational resilience (Le and Behl, 2023).

Furthermore, SMEs’ AI capabilities could directly impact organizational performance. Artificial intelligence systems enable organizations to automate mundane tasks, cut down on operating costs, streamline logistics management, and manage their customers’ relationships better. Prior literature indicates that organizations’ adoption of AI systems enhances not only the financial performance measures but also the non-financial performance indicators (Keong et al., 2025). Nevertheless, the effects of AI capabilities on SMEs’ performance may be contingent upon the strategic utilization of technological resources.

In Malaysia and Saudi Arabia, SMEs increasingly adopt AI technologies to support digital transformation and sustainable competitiveness. Nevertheless, differences in technological readiness and managerial capability continue to influence the effectiveness of AI implementation. SMEs with stronger AI capabilities are therefore expected to demonstrate higher strategic intelligence and improved organizational performance. Accordingly, this study proposes the following hypotheses:

*H3*: AI Capabilities positively impact Strategic Intelligence.

*H4*: AI Capabilities positively impact SME Performance.

### 2.3 Influence of risk management and innovativeness skills on entrepreneurial competency

Entrepreneurial competency is increasingly recognized as an important capability that enables SMEs to adapt to dynamic business environments and sustain long-term competitiveness. It includes the ability to identify opportunities, manage uncertainty, make strategic decisions, and develop innovative solutions. In SMEs, entrepreneurial competency is particularly important because firms often operate with limited resources and face greater exposure to environmental uncertainty and market volatility.

Risk management skills contribute significantly to entrepreneurial competency by enabling firms to identify, assess, and respond to potential threats and uncertainties (Falkner and Hiebl, 2015; Sagita et al., 2025). Effective risk management allows SME owners and managers to make informed decisions, allocate resources strategically, and improve organizational resilience. Resource Orchestration Theory (ROT) suggests that firms achieve competitive advantage when resources and capabilities are effectively coordinated to manage environmental challenges (Abdelwahed and Alshaikhmubarak, 2023). In rapidly changing business environments such as Malaysia and Saudi Arabia, SMEs with stronger risk management capabilities are more likely to improve adaptability and maintain competitive performance. Risk management also supports proactive strategic behavior by helping firms anticipate disruptions and respond effectively to emerging opportunities and threats.

Innovativeness skills represent another important component of entrepreneurial competency. These skills involve creative thinking, opportunity recognition, and the development of new ideas, products, or processes that improve organizational competitiveness. Previous studies indicate that innovation capability is a major driver of SME growth and sustainability because innovative firms are more capable of responding to changing customer demands and technological developments (Rhee et al., 2010). Core Competency Development Theory further explains that organizations develop competitive advantage through unique capabilities that differentiate them from competitors.

Recent studies suggest that innovative SMEs are more likely to adopt emerging technologies, improve operational processes, and strengthen strategic flexibility (Mikalef et al., 2022). Innovativeness also enhances entrepreneurs’ ability to identify market gaps and implement creative solutions that create value for customers and stakeholders. In Malaysia and Saudi Arabia, SMEs increasingly rely on innovation and digital transformation initiatives to strengthen competitiveness and sustainability within evolving economic environments.

Collectively, risk management and innovativeness skills strengthen entrepreneurial competency by improving organizational adaptability, strategic responsiveness, and opportunity recognition. SMEs that effectively combine these capabilities are therefore more likely to enhance entrepreneurial effectiveness and long-term business sustainability. Accordingly, this study proposes the following hypotheses:

*H5*: Risk Management Skills positively impact Entrepreneurial Competency.

*H6*: Innovativeness Skills positively impact Entrepreneurial Competency.

### 2.4 Entrepreneurial competency and its impact on strategic intelligence and SME performance

Entrepreneurial competency refers to the ability of entrepreneurs and managers to identify opportunities, make strategic decisions, manage resources, and adapt to changing business environments (Mohamad et al., 2025). These competencies are particularly important for SMEs because firms operating in competitive and uncertain markets require flexible leadership and innovative strategic capabilities to sustain growth. Entrepreneurial competency supports opportunity recognition, strategic planning, innovation, and organizational adaptability, which collectively strengthen business sustainability and competitiveness.

Strategic intelligence refers to the ability of organizations to collect, analyze, and use strategic information for planning purposes and competitive positioning. Entrepreneurial competency is an important determinant of strategic intelligence as competent entrepreneurs are able to do environmental scanning and analyze opportunities effectively (Imjai et al., 2024). Thus, SMEs that exhibit stronger entrepreneurial competency are able to foresee changes in the environment and respond to competitive challenges appropriately. According to Resource Orchestration Theory, strategic outcomes are possible due to orchestration and coordination of resources and capabilities of firms (Sirmon et al., 2011). Consequently, entrepreneurial competency helps SMEs manage their technological, financial, and human capital effectively.

Finally, it may be possible for entrepreneurs to positively affect performance of their SMEs by improving their own competencies. For example, according to Core Competency Development Theory, competitive advantage of firms is provided by managers and organizations in terms of distinctive managerial capabilities (Ismail et al., 2022). Thus, entrepreneurial competencies contribute to operational efficiency, innovation capabilities, and market orientation. Innovativeness, proactive attitude, and strategic flexibility allow entrepreneurs to detect and address inefficiencies and use technological advancements including artificial intelligence.

Previous studies have reported a positive relationship between entrepreneurial competency and SME performance in developing economies (Ismail et al., 2022). Entrepreneurially competent SMEs are generally more capable of improving operational effectiveness, financial performance, and long-term sustainability (Bag et al., 2021). In Malaysia and Saudi Arabia, SMEs increasingly depend on entrepreneurial leadership and digital adaptability to remain competitive within rapidly changing economic environments. Consequently, entrepreneurial competency represents a critical organizational capability that supports both strategic intelligence and SME performance. Accordingly, this study proposes the following hypotheses:

*H7*: Entrepreneurial Competency positively impacts Strategic Intelligence.

*H8*: Entrepreneurial Competency positively impacts SME Performance.

### 2.5 Impact of strategic intelligence on SME performance

Strategic intelligence is increasingly recognized as an important organizational capability that supports long-term competitiveness and sustainability among SMEs. It refers to the process of collecting, analyzing, and utilizing strategic information related to market trends, competitive conditions, stakeholder expectations, and environmental changes to support organizational decision-making (Imjai et al., 2024). For SMEs operating in dynamic and uncertain environments, strategic intelligence enables firms to improve adaptability, reduce uncertainty, and strengthen strategic planning.

SMEs frequently experience limited resource capacity and high levels of market instability, requiring sound decisions to enable their survival and development. The use of strategic intelligence will allow enterprises to discover new business opportunities, detect market disturbances, and react accordingly to market competition (Alam et al., 2022; Ojiako et al., 2015). In particular, through environmental scanning and competitive intelligence, SMEs can ensure that their business strategies correspond with customer trends and market changes. Such a practice will prove especially useful for SMEs operating in Malaysia and Saudi Arabia.

According to the Resource Orchestration Theory (ROT), firm performance depends on the ability of an organization to mobilize and coordinate its resources (Sirmon et al., 2011). Strategic intelligence plays a role in achieving this aim as it helps organizations leverage their informational resources to optimize strategic and operational activities. Past research demonstrates that strategic intelligence exerts a positive impact on various performance outcomes such as financial, innovation, efficiency, and competitive (Belhadi et al., 2024). At the same time, Mikalef and Gupta (2021) suggest that strategic intelligence allows firms to be more agile in adapting to changes.

Core Competency Development Theory further suggests that firms gain sustainable competitive advantage through specialized strategic and managerial capabilities (Prahalad and Hamel, 1990). Strategic intelligence enables SMEs to strengthen innovation, improve resource allocation, and enhance organizational resilience in uncertain business environments. SMEs that effectively utilize strategic intelligence are therefore more capable of sustaining long-term growth and competitiveness. Accordingly, this study proposes the following hypothesis:

*H9*: Strategic Intelligence positively impacts SME Performance.

## 3 Research methodology

### 3.1 Research design and sampling strategy

This study adopted a cross-sectional quantitative study to examine the effect of strategic intelligence on the performance of manufacturing SMEs in Malaysia and Saudi Arabia. According to Trochim and Donnelly (2008), A quantitative approach is appropriate for examining the relationships between variables in a positivist paradigm. The primary study goal was to understand the extent to which some constructs mediate the rise in SMEs’ performance in the manufacturing sector, particularly influencing this sector’s contribution to the economies of Malaysia and Saudi Arabia. SMEs in the manufacturing sector formed the target population as these companies not only have a high percentage of the nation’s GDP but also align with the objectives of economic diversification envisioned in Saudi Arabia’s Vision 2030. This study used purposive sampling. This sampling strategy enabled the selection of small and medium-sized enterprises that met specific predetermined criteria, such as being in existence for at least 12th and having decision-makers (i.e., owners or managers) with more than 3 years of strategic experience. Previous studies (Cheng and Kong, 2024) have established that purposive sampling can enhance the validity of research findings and the generalizability of results.

### 3.2 Data collection procedure

Data were collected from SMEs operating in major industrial regions of Malaysia and Saudi Arabia. Researchers collaborated with the Saudi Small and Medium Enterprises Authority (Monsha’at) to facilitate access to SME owners and managers. The questionnaire was distributed through targeted email communication to SME owners and managers identified with the support of Monsha’at. This approach ensured that only respondents who met the predefined purposive sampling criteria participated in the study. A total of 500 questionnaires were distributed, and 350 responses were received, representing a response rate of 70%. During the data screening process, 30 responses were excluded because they contained substantial missing values, incomplete questionnaire sections, or inconsistent response patterns. After data cleaning, the final dataset consisted of 320 valid responses, including 200 SMEs from Malaysia and 120 SMEs from Saudi Arabia. The study analyzed the data using a pooled sample approach to examine the overall relationships among the constructs across both developing economy contexts.

#### 3.2.1 Respondent profile

Table 1 presents the demographic profile of respondents from Malaysia and Saudi Arabia. The sample consisted of SME owners, managers, IT professionals, and business practitioners operating across multiple industrial sectors. Respondents were drawn from diverse demographic and organizational backgrounds, which supports the representativeness of the study within the selected developing economy contexts. A relatively higher proportion of respondents originated from the Information Technology (IT) sector, reflecting the increasing involvement of technology-oriented SMEs in AI adoption and digital transformation initiatives.

**Table 1:** Respondent profile by country.

Category	Subcategory	Malaysia (*n* = 200)	%	Saudi Arabia (*n* = 120)	%
Gender	Male	118	59.0%	74	61.7%
	Female	82	41.0%	46	38.3%
Age	20–29	42	21.0%	22	18.3%
	30–39	68	34.0%	44	36.7%
	40–49	60	30.0%	36	30.0%
	50 and above	30	15.0%	18	15.0%
Education	High school diploma	18	9.0%	14	11.7%
	Bachelor’s degree	102	51.0%	58	48.3%
	Master’s degree	60	30.0%	36	30.0%
	Doctorate	20	10.0%	12	10.0%
Position	Owner/manager/chief officer	52	26.0%	31	25.8%
	IT director	38	19.0%	23	19.2%
	Head of IT department	28	14.0%	17	14.2%
	Chief executive officer	18	9.0%	11	9.2%
	IT project manager	16	8.0%	10	8.3%
	Business analyst	16	8.0%	10	8.3%
	Other	32	16.0%	18	15.0%
Ownership type	Sole proprietorship	120	60.0%	72	60.0%
	Partnership/private ltd.	80	40.0%	48	40.0%
Sector type	Information technology (IT)	84	42.0%	56	46.7%
	Manufacturing	36	18.0%	20	16.7%
	Retail and wholesale	30	15.0%	18	15.0%
	Services	28	14.0%	14	11.7%
	Other	22	11.0%	12	10.0%
AI experience	Less than 1 year	48	24.0%	32	26.7%
	1–3 years	72	36.0%	40	33.3%
	3–5 years	40	20.0%	24	20.0%
	More than 5 years	40	20.0%	24	20.0%

Respondent distribution across the two countries offers an element of contextual diversity regarding the investigation of the capability and performance issues of SMEs within the developing economies framework. Sample Size Adequacy The sample size adequacy of the survey was calculated using standard structural equation modeling (SEM) principles. As noted by Tinsley and Tinsley (1987), A sample size from 5 to 10 observations per indicator is considered adequate for SEM analysis. With 37 indicators used in the present investigation, the required sample size would be in range between 185 and 370 respondents. Moreover, according to Li and Lomax (2017), a general rule of thumb suggests that sample sizes between 100 and 500 respondents are generally required for SEM studies. A g*power analysis showed that at least 185 respondents were needed to ensure a reasonable amount of statistical power for this research study.

### 3.3 Measurement instruments

The constructs in this study were measured using a five-point Likert scale ranging from 1 (“strongly disagree”) to 5 (“strongly agree”). All questionnaire items were adapted from previously validated scales to ensure content validity and measurement reliability. AI Capabilities were adapted from Mikalef and Gupta (2021), while AI Integration Skills and Smart Decision-Making Skills were adapted from prior studies on AI adoption and digital decision-making capabilities (Al-Rahmi, 2025). Risk Management Skills and Innovativeness Skills were adapted from entrepreneurial competency and innovation (Sagita et al., 2025). Entrepreneurial Competency items were adapted from (Okolie et al., 2021; Sakib et al., 2022). Strategic Intelligence was measured using a five-item scale adapted from (Mahmud et al., 2025; Nenzhelele and Pellissier, 2014), which includes dimensions such as environmental scanning and knowledge management. SME Performance was measured using a five-item scale adapted from (Al Koliby et al., 2024; Wiklund and Shepherd, 2003). The Cronbach’s alpha values for all constructs exceeded the recommended threshold of 0.70, indicating satisfactory internal consistency and reliability. To minimize potential confounding effects, firm size, firm age, and industry type were included as control variables in the analysis.

### 3.4 Analytical methodology

Before conducting the structural equation modeling analysis, data normality was assessed using skewness and kurtosis statistics. Since the study employed a five-point Likert scale, a perfect normal distribution was not expected. However, according to Hair et al. (2019) and Kline (2015), Skewness values within ±2 and kurtosis values within ±7 are considered acceptable for SEM analysis. The results indicated that all construct items fell within the recommended threshold ranges, confirming the absence of severe non-normality in the dataset. Nevertheless, PLS-SEM was considered more appropriate for this study because it is a variance-based, non-parametric approach that performs well with complex models, exploratory research designs, and data that may not fully satisfy multivariate normality assumptions.

Based on Hair and Alamer (2022) and Sarstedt et al. (2021). The current research used PLS-SEM via SmartPLS 4.0 because it could examine complex models that have multiple latent constructs and indicators. Unlike covariance-based SEM (CB-SEM) and regression, which require normal distribution and larger samples, PLS-SEM is optimally useful for exploratory research and hierarchical models, even if the data are non-normal. The current situation shows that the newly developed method is successful, and the researchers have managed to avoid side effects in their study. PLS-SEM was the best choice for methodical rigor and actionable results. This strategy is particularly relevant for exploratory studies in new contexts such as Malaysia and Saudi Arabia. The analytical technique adopted for the two-stage process involved measurement model assessment, whose reliability and validity of the constructs were assessed using internal consistency (Cronbach’s alpha and composite reliability), convergent validity (average variance extracted, AVE), and discriminant validity (HTMT and Fornell–Larcker Criterion). The posited relationships were tested using path coefficients, *t*-statistics, and *p*-values. *R*^2^ was used to assess the predictive ability of the model.

## 4 Results and analysis

### 4.1 Measurement model

The measurement model was assessed using factor loadings, internal consistency reliability, convergent validity, discriminant validity, and descriptive statistics. Factor loadings were extracted directly from the SmartPLS 4.0 output during the assessment of the reflective measurement model. Most factor loading values exceeded the recommended threshold of 0.70 (Hair et al., 2019), indicating satisfactory indicator reliability. One item (AIC05 = 0.68) was slightly below the recommended threshold but was retained because the overall construct reliability and convergent validity remained acceptable. The factor loading results are presented in Table 2. AI Capabilities demonstrated loadings ranging from 0.68 to 0.82, while AI Integration Skills ranged from 0.75 to 0.85. Entrepreneurial Competency exhibited loadings between 0.77 and 0.87, indicating strong construct validity. Innovativeness Skills and Smart Decision-Making Skills also demonstrated strong loadings above 0.80. Similarly, Strategic Intelligence and SME Performance showed satisfactory item reliability, with loadings ranging from 0.78 to 0.85. Overall, the results confirmed acceptable convergent validity and reliability of the measurement model, supporting its suitability for subsequent structural model assessment.

**Table 2:** Measurement model (factor loading).

Constructs	Items	Factor loadings	VIF	Constructs	Items	Factor loadings	VIF
AI capabilities	AIC01	0.79	1.76	Risk management skills	RMS01	0.82	1.81
	AIC02	0.82	2.02		RMS02	0.83	1.90
	AIC03	0.81	2.09		RMS03	0.84	1.90
	AIC04	0.76	1.64		RMS04	0.72	1.40
	AIC05	0.68	1.38		SDMS01	0.86	2.28
AI integration skills	AIIS01	0.75	1.63	Smart decision-making skills	SDMS02	0.84	2.22
	AIIS02	0.85	2.25		SDMS03	0.87	2.50
	AIIS03	0.82	1.99		SDMS04	0.86	2.18
	AIIS04	0.77	1.74		SIN01	0.78	1.83
	AIIS05	0.79	1.94		SIN02	0.79	1.79
Entrepreneurial competency	EC01	0.79	1.78	Strategic intelligence	SIN03	0.82	2.11
	EC02	0.79	1.91		SIN04	0.83	2.36
	EC03	0.87	2.48		SIN05	0.8	2.00
	EC04	0.82	2.12		SMP01	0.84	2.42
	EC05	0.77	1.79		SMP02	0.85	2.59
Innovativeness skill	IS01	0.83	1.93	SMEs performance	SMP03	0.84	2.26
	IS02	0.84	2.07		SMP04	0.82	2.3
	IS03	0.85	2.14		SMP05	0.78	1.95
	IS04	0.85	2.05				

We also assess the common method bias (CMB) and multicollinearity. This study employed Harman’s single-factor test and the full collinearity variance inflation factor (VIF) approach recommended for PLS-SEM studies (Kock, 2021). The VIF values presented in Table 2 ranged from 1.63 to 2.59, which are below the recommended threshold of 3.3, indicating that common method bias and multicollinearity are unlikely to threaten the validity of the findings. In addition, Harman’s single-factor test revealed that the first factor accounted for 37.00% of the total variance, which is below the commonly accepted threshold of 50% (Podsakoff et al., 2003). Both methods provide additional assurance regarding the robustness of the measurement model and reduce concerns related to common method variance.

The reliability analysis results indicated that all the constructs had a high internal consistency, as shown by the Cronbach’s *α* value of the constructs ranging between 0.81 (RMS) and 0.89 (SMP) and the CR value of the constructs ranging between 0.88 (AIC, RMS) and 0.92 (SDMS, SMP), which all surpassed the minimum threshold value of greater than 0.70. The AVE of AIC indicates a value of 0.60; SDMS indicates a value of 0.74. These values were higher than the cut-off value of 0.50, as shown in Table 3. Thus, convergent validity was also supported. Leading components such as AI Capabilities, Entrepreneurial Competency, and SME Performance showed very high reliability and validity with *α* = 0.83, CR = 0.88, AVE = 0.60; *α* = 0.87, CR = 0.90, AVE = 0.65, and *α* = 0.89, CR = 0.92, AVE = 0.69, respectively. The results in Table 3 confirm the stability of the measurement model, which can be used as the basis for further structural analysis.

**Table 3:** Reliability tests (alpha, CR, and AVE).

Constructs	Mean	SD	Cronbach’s alpha	Composite reliability	Average variance extracted
AIC	3.89	0.72	0.83	0.88	0.60
AIIS	3.95	0.69	0.86	0.9	0.64
EC	4.01	0.66	0.87	0.9	0.65
IS	3.91	0.71	0.86	0.91	0.71
RMS	3.87	0.74	0.81	0.88	0.64
SDMS	3.98	0.68	0.88	0.92	0.74
SIN	3.93	0.70	0.86	0.9	0.65
SMP	3.88	0.73	0.89	0.92	0.69

The descriptive statistics presented in Table 3 indicate that respondents generally reported moderate to high perceptions across all study constructs. The mean values ranged from 3.87 to 4.01, suggesting that respondents demonstrated relatively positive perceptions regarding AI capabilities, entrepreneurial competency, strategic intelligence, and SME performance. Entrepreneurial Competency (EC) recorded the highest mean score (*M* = 4.01, SD = 0.66), followed by Smart Decision-Making Skills (SDMS) (*M* = 3.98, SD = 0.68) and AI Integration Skills (AIIS) (*M* = 3.95, SD = 0.69). In contrast, Risk Management Skills (RMS) showed the lowest mean score (*M* = 3.87, SD = 0.74), although the value still reflected a moderately positive perception among respondents. The relatively low standard deviation values across all constructs indicate acceptable consistency in respondents’ evaluations and suggest limited variability within the dataset.

Moreover, we also employed the HTMT and Fornell–Larcker criteria to assess discriminant validity among the study constructs, as presented in Tables 4 and 5. Most HTMT values were below the conservative threshold of 0.85 recommended by Henseler et al. (2015), with a few closely related constructs showing slightly higher values. For example, Entrepreneurial Competency and Risk Management Skills demonstrated an HTMT value of 0.89. However, all HTMT values remained below the liberal threshold of 0.90, indicating acceptable discriminant validity (Dahri et al., 2024; Fornell and Larcker, 1981; Henseler et al., 2015). Furthermore, the findings suggest that although several constructs are conceptually related within the proposed framework, the measurement model maintains sufficient discriminant validity for structural model assessment.

**Table 4:** Discriminant validity HTMT.

Constructs	AIC	AIIS	EC	IS	RMS	SDMS	SIN	SMP
AIC								
AIIS	0.85							
EC	0.71	0.69						
IS	0.62	0.67	0.83					
RMS	0.76	0.73	0.89	0.89				
SDMS	0.66	0.61	0.75	0.76	0.87			
SIN	0.66	0.67	0.79	0.8	0.83	0.7		
SMP	0.66	0.66	0.78	0.81	0.87	0.76	0.76	

**Table 5:** Discriminant validity Fornell–Larcker criterion.

Constructs	AIC	AIIS	EC	IS	RMS	SDMS	SIN	SMP
AIC	0.77							
AIIS	0.71	0.8						
EC	0.6	0.6	0.81					
IS	0.53	0.58	0.72	0.84				
RMS	0.62	0.61	0.74	0.75	0.8			
SDMS	0.56	0.53	0.66	0.66	0.74	0.86		
SIN	0.56	0.58	0.69	0.69	0.7	0.61	0.81	
SMP	0.57	0.58	0.69	0.71	0.74	0.67	0.66	0.83

The Fornell–Larcker criterion provided discriminant validity, as the square root of the Average Variance Extracted (AVE) for every construct was greater than its correlation with other constructs. For instance, the square root of AVE for AI Capabilities stood at 0.77, more than the correlation with AI Integration Skills, which was at 0.71, and Entrepreneurial Competency, at 0.6. Similarly, SME Performance demonstrated a strong square root of AVE at 0.83, greater than the correlations with the other constructs under study, including SIN = 0.66. These results indicate that all constructs were well differentiated, thus validating the measurement model.

### 4.2 Structural model

The structural model was assessed using model fit indices, coefficient of determination (*R*^2^), and effect size analysis (*f*^2^) following PLS-SEM guidelines. The model fit was assessed using the standardized root mean square residual (SRMR), normed fit index (NFI), and RMS theta, following the recommendations for PLS-SEM analysis (Hair et al., 2019). The SRMR value was 0.067, which is below the recommended threshold of 0.08, indicating an acceptable model fit. In addition, the NFI value of 0.901 demonstrated satisfactory model adequacy. The RMS theta value of 0.109 was also within the acceptable threshold, further supporting the reliability of the reflective measurement model. The model fit indices confirm that the proposed structural model adequately represents the observed data.

According to Hair J. et al. (2017), *R*^2^ values indicate the proportion of variance in endogenous constructs explained by the exogenous variables in the structural model. Table 6 shows that AI Capabilities (AIC) and SME Performance (SMP) demonstrated good explanatory power with *R*^2^ values of 0.56. This indicates that AI Integration Skills, Smart Decision-Making Skills, Strategic Intelligence, and Entrepreneurial Competency meaningfully explain variations in organizational outcomes. Entrepreneurial Competency (EC) showed the highest explanatory power with an *R*^2^ value of 0.61, highlighting the importance of entrepreneurial capabilities in supporting strategic transformation and innovation among SMEs (Khan et al., 2021; Soomro et al., 2024, 2025). Strategic Intelligence (SIN) also demonstrated moderate explanatory power with an *R*^2^ value of 0.50, supporting its role as an important mediating construct linking competencies to SME performance outcomes. Furthermore, the adjusted *R*^2^ values were slightly lower than the corresponding *R*^2^ values across all endogenous constructs, confirming the stability and robustness of the structural model (Hair J. et al., 2017). The findings support the argument that AI capabilities and entrepreneurial competencies function as strategic enablers that strengthen SME competitiveness and organizational performance in digitally transforming business environments.

**Table 6:** *R*-square values.

Constructs	*R*-square	*R*-square adjusted
AIC	0.560	0.557
EC	0.610	0.607
SIN	0.500	0.496
SMP	0.560	0.554

The effect size analysis (*f*^2^) highlights the relative contribution of each exogenous construct to its corresponding endogenous construct, as presented in Table 7. AI Integration Skills (AIIS) demonstrated a substantial effect on AI Capabilities (AIC) (*f*^2^ = 0.54), indicating that technical integration competencies are essential for strengthening organizational AI capabilities in SMEs. Similarly, Entrepreneurial Competency (EC) showed a strong effect on Strategic Intelligence (SIN) (*f*^2^ = 0.39) and a moderate effect on SME Performance (SMP) (*f*^2^ = 0.14), emphasizing the strategic importance of entrepreneurial capabilities in enhancing organizational outcomes. Risk Management Skills (RMS) and Innovativeness Skills (IS) also exhibited moderate effects on Entrepreneurial Competency, with *f*^2^ values of 0.24 and 0.16, respectively. In addition, Smart Decision-Making Skills (SDMS) had a moderate contribution to AI Capabilities (*f*^2^ = 0.11), suggesting that strategic decision-making supports AI capability development.

**Table 7:** *F*-squared values.

Paths	*f* ^2^
AIC → SIN	0.070
AIC → SMP	0.040
AIIS → AIC	0.540
EC → SIN	0.390
EC → SMP	0.140
IS → EC	0.160
RMS → EC	0.240
SDMS → AIC	0.110
SIN → SMP	0.110

Although AI Capabilities significantly influenced SME Performance, the effect size was relatively weak (*f*^2^ = 0.04). This finding suggests that AI capabilities alone may not be sufficient to generate substantial performance improvements in SMEs unless they are supported by complementary organizational capabilities such as strategic intelligence, entrepreneurial competency, and effective resource orchestration. In resource-constrained SME environments, the business value of AI is often realized indirectly through improved strategic decision-making, adaptability, and organizational intelligence rather than through direct operational effects alone (Parnell et al., 2015; Yi et al., 2023). The findings therefore support the argument that AI technologies should be integrated with human and strategic capabilities to achieve meaningful organizational performance outcomes.

Results of Hypothesis Testing Table 8 shows the results of the hypotheses tests. Statistical inference requires *p*-values to show the probability of obtaining the observed result if the null hypothesis holds true, while *t*-values represent the degree of strength and direction of the relationship between variables. In this case, the obtained results have shown high robustness, in support of all proposed relationships with the threshold values of *p*-values being larger than 0.01 and *t*-statistics larger than 1.96 (Hair J. et al., 2017). AI Integration Skills and Smart Decision-Making Skills positively affected AI Capabilities (*β* = 0.58, *t* = 11.14; *β* = 0.26, *t* = 5.07, respectively), suggesting that these abilities play a key role in acquiring related capabilities. Further, AI Capabilities had a positive effect on Strategic Intelligence (*β* = 0.23, *t* = 4.33) and SME performance (*β* = 0.18, *t* = 4.06), thereby proving the importance of these capabilities for SME performance. Risk Management Skills (*β* = 0.46, *t* = 6.89) and Innovativeness Skills (*β* = 0.37, *t* = 5.43) significantly impacted Entrepreneurial Competency and therefore played an important part in innovation and adaptability. Furthermore, Entrepreneurial Competency proved to be the main driver of both Strategic Intelligence (*β* = 0.55, *t* = 9.39) and SME performance (*β* = 0.36, *t* = 5.94). Lastly, Strategic Intelligence significantly impacted SME performance (*β* = 0.32, *t* = 5.06), meaning that this factor was important in improving organizational performance. These findings were supported by similar conclusions about the importance of AI capabilities (Abou-Foul et al., 2023), strategic intelligence (Goswami, 2020), and entrepreneurial competency (Imjai et al., 2024) in relation to firm performance. Consequently, further improvement in integrating AI, improving decision-making abilities, and increasing entrepreneurship in SMEs is needed to enhance firm performance.

**Table 8:** Hypothesis testing (direct).

Hypothesis	Original sample	*t* statistics	*p* values	Decision
AIC → SIN	0.23	4.33	0.00	Supported
AIC → SMP	0.18	4.06	0.00	Supported
AIIS → AIC	0.58	11.14	0.00	Supported
EC → SIN	0.55	9.39	0.00	Supported
EC → SMP	0.36	5.94	0.00	Supported
IS → EC	0.37	5.43	0.00	Supported
RMS → EC	0.46	6.89	0.00	Supported
SDMS → AIC	0.26	5.07	0.00	Supported
SIN → SMP	0.32	5.06	0.00	Supported

**Figure 3:**
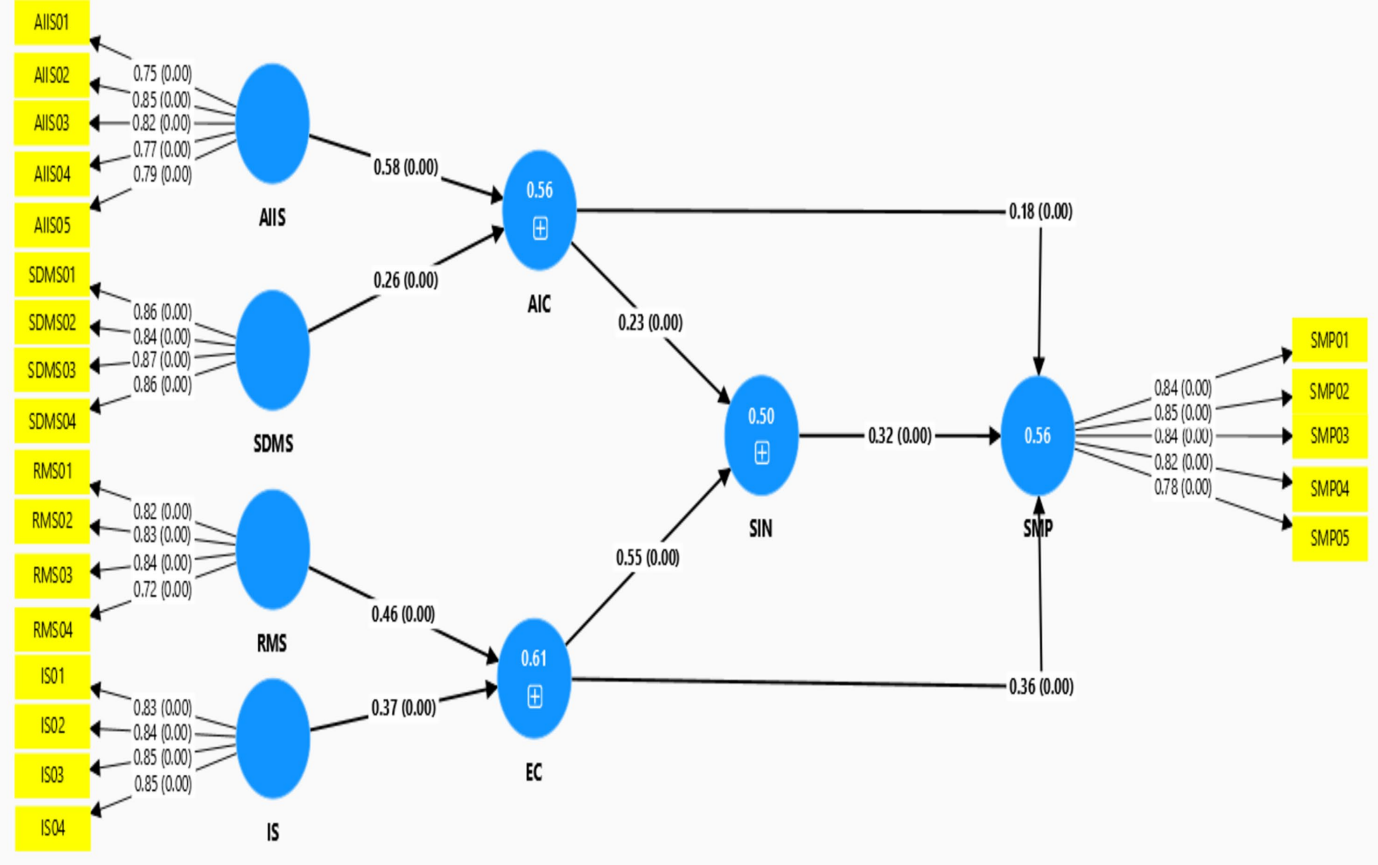
Hypothesis results.

The indirect relationships were tested, and the results are presented in Table 9 and Figure 3, and all the hypotheses formulated were confirmed. This is evidence that significant mediating effects exist. It was also observed that AIC, SIN, and SMP were significantly related, with *β* = 0.07, *t* = 3.24, and *p* < 0.01. Thus, it has been proven that strategic intelligence mediates AI capabilities and SMEs’ performance. Comparably, the relationship between Entrepreneurial Competency (EC), Strategic Intelligence (SIN), and SME performance (SMP) was also noteworthy (*β* = 0.17, *t* = 4.08, *p* < 0.01), thereby validating the mediating function of strategic intelligence. For the mediating effect of AI capabilities, AI Integration Skills (AIIS) → AI Capabilities (AIC) → Strategic Intelligence (SIN) had a strong mediation with *β* = 0.13, *t* = 4.34, *p* < 0.01, and AI Integration Skills → AI Capabilities → SMEs Performance (SMP) was similarly significant with *β* = 0.10, *t* = 3.99, *p* < 0.01. Similarly, Innovativeness Skills (IS) → Entrepreneurial Competency (EC) → Strategic Intelligence (SIN) (*β* = 0.21, *t* = 4.50, *p* < 0.01) and IS → EC → SMEs Performance (*β* = 0.14, *t* = 3.95, *p* < 0.01) were strongly mediated. Risk management competencies were found to be critical mediators through the pathways of RMS to EC to SIN (*β* = 0.25, *t* = 5.41, *p* < 0.01) and RMS to EC to SMP (*β* = 0.17, *t* = 4.25, *p* < 0.01), which showed significant indirect effects. Similarly, Smart Decision-Making Skills (SDMS) → AI capabilities (SIN) (*β* = 0.06, *t* = 2.84, *p* < 0.01) and SDMS → AI capabilities → SMEs performance (SMP) (*β* = 0.05, *t* = 2.80, *p* < 0.05) revealed significant mediation. Presence of more complex multi-path mediations, particularly SDMS → AIC → SIN → SMP with *β* = 0.02, *t* = 2.46 and *p* < 0.05, IS → EC → SIN → SMP with *β* = 0.07, *t* = 3.08 and *p* < 0.01, AIIS → AIC → SIN → SMP with *β* = 0.04, *t* = 3.24 and *p* < 0.01, and RMS → EC → SIN → SMP with *β* = 0.08, *t* = 3.53 and *p* < 0.01. All the *t*-values exceeded the critical value of 1.96 at *p* < 0.05, and most of the cases had *p*-values less than 0.01 (Hair J. et al., 2017; Hair J. F. Jr et al., 2017). This shows that strategic intelligence, entrepreneurial competency, and AI capabilities are strong mediators of better SME performance.

**Table 9:** Hypothesis testing (indirect).

Relationships	Original sample	*t* statistics	*p* values	Decision
AIC → SIN → SMP	0.07	3.24	0.00	Supported
EC → SIN → SMP	0.17	4.08	0.00	Supported
AIIS → AIC → SIN	0.13	4.34	0.00	Supported
IS → EC → SIN	0.21	4.5	0.00	Supported
AIIS → AIC → SMP	0.1	3.99	0.00	Supported
IS → EC → SMP	0.14	3.95	0.00	Supported
RMS → EC → SIN	0.25	5.41	0.00	Supported
RMS → EC → SMP	0.17	4.25	0.00	Supported
SDMS → AIC → SIN	0.06	2.84	0.00	Supported
SDMS → AIC → SMP	0.05	2.8	0.01	Supported
SDMS → AIC → SIN → SMP	0.02	2.46	0.01	Supported
IS → EC → SIN → SMP	0.07	3.08	0.00	Supported
AIIS → AIC → SIN → SMP	0.04	3.24	0.00	Supported
RMS → EC → SIN → SMP	0.08	3.53	0.00	Supported

## 5 Discussion

AI is developing at such a rapid pace that it is being used by almost all organizations, especially small and medium-sized enterprises. The relationship between AI capabilities, business skills, strategic knowledge, and SMEs’ performance was investigated using a powerful structural equation model (SEM). The results indicate that AI capabilities can mediate the effect of entrepreneurial competency and others on improving the performance and strategic intelligence of SMEs. The results agree with the rapidly growing evidence that AI technology will transform organizational decisions and performance (Chatterjee et al., 2022).

### 5.1 AI capabilities

The findings indicate that AI integration skills play an important role in strengthening AI capabilities among SMEs. The results suggest that SMEs require not only access to AI technologies but also the organizational ability to integrate these technologies into operational and strategic activities. This finding is consistent with recent studies arguing that AI adoption becomes more effective when firms possess sufficient technical integration capability, digital readiness, and managerial support (Shamim et al., 2023). In the contexts of Malaysia and Saudi Arabia, SMEs increasingly face pressure to adopt digital technologies to remain competitive in rapidly evolving markets. Therefore, AI integration capability appears to be an important strategic resource for improving organizational adaptability and decision-making (Abdelwaheed et al., 2025; Khan et al., 2020).

The findings further indicate that AI capabilities contribute to the development of strategic intelligence. This suggests that AI technologies can support SMEs in improving market forecasting, identifying business opportunities, and enhancing strategic responsiveness. Prior literature similarly emphasizes that AI-driven analytics assist organizations in converting large volumes of data into actionable insights that improve strategic planning and organizational agility (Al Koliby et al., 2024). For SMEs operating in developing economies, AI capabilities may help compensate for limitations in traditional managerial and analytical resources by improving evidence-based decision-making processes.

Although AI capabilities positively influenced SME performance, the effect size remained relatively weak, suggesting that AI adoption alone may not automatically generate substantial organizational performance improvements. This finding indicates that SMEs in Malaysia and Saudi Arabia may still face structural, financial, and managerial challenges in translating AI investments into measurable business outcomes. In many SMEs, AI implementation remains constrained by limited technical expertise, insufficient digital infrastructure, and uncertainty regarding long-term returns from AI adoption. Therefore, AI capabilities appear to contribute more effectively when combined with complementary organizational capabilities such as strategic intelligence and entrepreneurial competency. This finding aligns with recent studies emphasizing that technological resources alone are insufficient unless organizations possess the managerial and strategic capacity to integrate AI into decision-making and operational processes (Badghish and Soomro, 2024; Chuan-Fu and Kuppusamy, 2025).

### 5.2 Entrepreneurial competency

The findings indicate that entrepreneurial competency plays a central role in strengthening both strategic intelligence and SME performance. The results suggest that innovativeness and risk management capability are important dimensions of entrepreneurial competency that enable SMEs to adapt to dynamic business environments and identify emerging market opportunities. This finding is consistent with prior studies emphasizing that entrepreneurial competency enhances organizational flexibility, innovation capability, and strategic responsiveness in rapidly changing markets (Mukhopadhyay et al., 2024).

The findings further suggest that SMEs with stronger entrepreneurial competency are better positioned to manage uncertainty, respond proactively to competitive pressures, and sustain long-term organizational growth. In the contexts of Malaysia and Saudi Arabia, where SMEs increasingly operate under digital transformation pressures and economic uncertainty, entrepreneurial competency appears to function as an important strategic capability supporting business resilience and adaptability (Mohamad et al., 2025; Satar et al., 2025). The ability to combine innovation orientation with effective risk management may therefore strengthen SME competitiveness in developing economy environments.

Another key finding in this study is that it supports the view that entrepreneurial competency plays an important role in building strategic intelligence. The reason for this is that SMEs headed by entrepreneurial, competent leaders have the ability to detect environmental shifts, predict potential problems, and match their resources to strategic objectives. This finding is consistent with the dynamic capabilities theory in that the presence of entrepreneurial leadership improves the ability of organizations to adapt and detect uncertainties in the environment (Schoemaker et al., 2018).

### 5.3 Strategic intelligence

The findings confirm that strategic intelligence plays a critical role in enhancing SME performance and acts as an important mechanism linking AI capabilities and entrepreneurial competency with organizational outcomes. SMEs with stronger strategic intelligence are better able to anticipate market changes, allocate resources effectively, and make evidence-based strategic decisions in highly dynamic business environments. This finding is consistent with prior studies emphasizing that strategic intelligence strengthens organizational adaptability, proactive decision-making, and long-term competitiveness (Satar et al., 2025).

This implies that strategic intelligence would be more effective through the combined influence of artificial intelligence abilities and entrepreneurship skills. Artificial intelligence abilities help organizations generate analysis while entrepreneurship skills help Small and Medium Enterprises utilize the information derived from such analyses for the creation of viable business strategies (Alhammadi, 2025). This means that there is a clear need for synergy between the two elements mentioned above.

In the context of Malaysia and Saudi Arabia, where SMEs are now experiencing the pressure to undergo digital transformation, economic instability, and fierce competition, strategic intelligence can be seen as one of the vital capabilities for ensuring business sustainability and performance (Aloulou, 2024). This means that the results support the claim that SMEs need more than just technology usage but must also develop their strategic and management capabilities to properly use artificial intelligence-driven information for decision-making.

### 5.4 Theoretical contribution

This study contributes to the growing literature on AI capabilities, entrepreneurial competency, strategic intelligence, and SME performance in developing economies. Grounded in Resource Orchestration Theory and dynamic capability perspectives, the study explains how technological and entrepreneurial capabilities jointly contribute to strategic intelligence and organizational performance. The findings demonstrate that AI capabilities function as important strategic resources that support organizational adaptability and competitive performance when combined with entrepreneurial competency and strategic intelligence.

The study further contributes to the literature by empirically validating the mediating role of strategic intelligence in the relationship between AI capabilities and SME performance. The findings suggest that technological capabilities alone may not directly generate substantial performance improvements unless organizations possess complementary strategic and managerial capabilities.

Moreover, this study adds value to the body of knowledge on SMEs and AI by offering empirical findings based on the Malaysian and Saudi Arabian contexts, where there is increasing adoption of digital technologies and resource constraints. This study provides an integrated perspective on the role of technological and human capabilities in enhancing the competitiveness and performance of SMEs.

### 5.5 Practical contribution

The results of this study have significant implications for SME stakeholders and policymakers in developing economies. There is a strong rationale for developing AI integration skills and entrepreneurial competencies in SMEs. Policymakers and business support agencies must design customized training programmes tailored to specific sectors to build advanced AI technical capabilities, such as AI-based inventory management, forensic prediction of customer behavior, automated preparation of financial statements, and soft managerial skills for project planning and overseeing AI adoption in SMEs for effective utilization. Strategic intelligence enables superior organizational performance. To make decisions and plan for the long term, SMEs must invest in tools and systems that help them analyze data. Officials can offer money for AI use and platforms for SMEs to swap knowledge to help SMEs adopt technology.

The study also suggests that specific support systems for enhancing entrepreneurial skills must be established. Training courses on risk management and innovation for SME owners and managers will assist in making better resource-allocation decisions under resource-constrained conditions. To help SMEs test and scale AI solutions, there is a need to set up entrepreneurial accelerators, AI-focused business incubators and sector-specific innovation hubs.

In addition, the SME sector can better adapt to changing demand through the innovative culture of the government. In addition, according to Saudi Vision 2030, which identifies digital transformation and SME empowerment as key drivers of national development, the government should ramp up efforts to address financial and regulatory barriers to AI adoption within SMEs. These include simplifying business license processes for AI, reducing import duties on AI hardware and software, and providing small and medium businesses with greater access to AI R&D grants. Policies aimed at enhancing competitiveness within the SME sector are consistent with the national objective of creating a digitally enabled economy.

The study suggests that SME (small and medium enterprises) development should be a multi-stakeholder initiative. This means the collaboration of the government, associations, and financial institutions to help SMEs overcome economic and regulatory challenges. As a result of streamlining loan processes, lowering interest rates, and government guarantees, SMEs will invest in appropriate technology and grow their businesses. In this sense, the present study delivers practical lessons to enhance the development and performance of SMEs in emerging economies.

## 6 Conclusion

This study examined the relationships among AI integration skills, smart decision-making skills, innovativeness skills, risk management skills, entrepreneurial competency, AI capabilities, strategic intelligence, and SME performance in the contexts of Malaysia and Saudi Arabia. Grounded in Resource Orchestration Theory, the study demonstrated that AI capabilities and entrepreneurial competency function as important strategic resources that enhance organizational adaptability, strategic intelligence, and SME performance in developing economy environments.

The findings suggest that AI technologies alone may not be sufficient to generate substantial organizational performance improvements unless they are supported by complementary managerial and strategic capabilities. In particular, strategic intelligence emerged as an important mechanism through which AI capabilities and entrepreneurial competency contribute to organizational performance. The study, therefore, highlights the importance of integrating technological capability, entrepreneurial orientation, and strategic decision-making to strengthen SME competitiveness and long-term business sustainability.

The results are particularly relevant in the context of Malaysia and Saudi Arabia, where SMEs are facing increasing pressure to digitally transform against the backdrop of economic uncertainties and changing competitive environments. SMEs functioning in these contexts could strengthen AI integration capability, innovation orientation, and strategic adaptability to enhance goods and services resilience and market responsiveness.

The findings of the study add to the body of knowledge on the development of organizational capabilities enabled by AI. The study adds to evidence that SME performance is sustainable not only where technology adoption takes place but also where human, strategic and technological resources are coordinated effectively.

### 6.1 Limitations and future work

The current research on strategic intelligence, entrepreneurial competency, and AI capabilities in Malaysia and Saudi Arabia has several limitations. These limitations include all mediating factors that influence the performance of SMEs in Malaysia and Saudi Arabia. The data may not accurately reflect reality, as they are self-reported. There may be biases due to social desirability. Future studies should include objective performance metrics or a multi-source data approach to enhance validity. Therefore, the data collection was cross-sectional and could not capture the temporal dynamics between the constructs. Long-lasting future studies may help confirm the process of this relationship.

Another limitation of this study is that the model was estimated using pooled data from Malaysia and Saudi Arabia without conducting multi-group analysis (MGA). Although both countries represent developing economy contexts, differences in institutional structures, regulatory environments, and cultural dimensions may influence SME adoption of AI technologies and strategic intelligence practices. Future research should apply MGA techniques to examine whether the proposed relationships differ significantly across national contexts.

This study used quantitative methods. PLS-SEM can successfully model both direct and indirect linear associations. However, it cannot capture complex and nonlinear interactions. The simplistic linearity of the model seems to miss any quadratic effect or moderation. PLS-SEM is less suited for estimating complex relationships than covariance-based SEM. It also has no advanced fit index. To overcome this issue, it is suggested that the PLS-SEM method should be used along with other procedures. Future studies can employ the latest AI models, such as ANN, and Explainable AI (XAI) for a detailed analysis.

Additionally, SMEs from Malaysia and Saudi Arabia were employed in the study, which limits the scope of the study to be generalizable to other sectors. Another limitation relates to the sectoral composition of the sample. Approximately 43.8% of respondents were drawn from the Information Technology (IT) sector, which may limit the generalizability of the findings to SMEs operating in non-technology-intensive industries. Since IT firms are more likely to possess higher digital readiness and AI familiarity, the observed relationships between AI capabilities, strategic intelligence, and SME performance may appear stronger than in more traditional sectors. Future research should examine more balanced cross-sector samples to validate the applicability of the proposed model across diverse SME industries.

The study did not consider policies and other contextual conditions of a country or cultural dimensions; thus, SME performance would also depend on that context. Future studies on SMEs should consider the contextual variables that evolve into more holistic and panoramic determinants of success across a range of settings.

In addition, no empirical evaluation was conducted on the possible moderating effects of firm size, industry sector, and organizational readiness. It is important to note such heterogeneity, even if the research does not focus on it. These moderators should be examined in future studies to identify new relationships and improve the model.

## Data Availability

The data that support the findings of this study are available from the corresponding author upon reasonable request.

## Appendix A: Survey questionnaire

**Table:**

Construct name	Item#	Items
AI Capabilities (AIC)	AIC01	Our organization effectively utilizes AI tools to optimize operations.
	AIC02	AI capabilities have improved decision-making processes in our organization.
	AIC03	Our team is proficient in implementing AI technologies across departments.
	AIC04	AI tools have enhanced the efficiency of business operations.
	AIC05	The adoption of AI has significantly impacted our strategic objectives.
AI Integration Skills (AIIS)	AIIS01	We possess the skills to integrate AI into existing workflows.
	AIIS02	Our organization regularly trains employees in AI technologies.
	AIIS03	AI integration has improved our operational flexibility.
	AIIS04	AI systems are seamlessly integrated into daily operations.
	AIIS05	Our organization is well-equipped to handle challenges in AI integration.
Entrepreneurial Competency (EC)	EC01	Our leaders demonstrate entrepreneurial capabilities to innovate using AI.
	EC02	We continuously strive to identify new opportunities through AI.
	EC03	Risk-taking is encouraged within our organization to innovate.
	EC04	Our team collaborates effectively to solve business challenges using AI.
	EC05	Entrepreneurial competency plays a significant role in our performance.
Innovativeness Skill (IS)	IS01	AI tools have fostered innovation within our organization.
	IS02	We encourage creative thinking in leveraging AI for business growth.
	IS03	AI integration has resulted in innovative solutions to complex problems.
	IS04	Our organization is a leader in adopting innovative AI practices.
Risk Management Skills (RMS)	RMS01	Our organization effectively uses AI to identify potential risks.
	RMS02	AI has enhanced our ability to manage business uncertainties.
	RMS03	Risk management is integrated into our AI implementation strategies.
	RMS04	AI tools are instrumental in mitigating operational risks.
Smart Decision-Making Skills (SDMS)	SDMS01	AI contributes significantly to strategic decision-making.
	SDMS02	Data-driven AI insights improve the quality of our decisions.
	SDMS03	AI tools have enhanced decision-making speed and accuracy.
	SDMS04	Decision-making processes are aligned with AI-generated recommendations.
Strategic Intelligence (SIN)	SIN01	AI has significantly contributed to our strategic foresight.
	SIN02	Strategic intelligence has improved with the integration of AI tools.
	SIN03	AI-driven intelligence supports our long-term business goals.
	SIN04	We utilize AI tools to adapt strategically to market changes.
	SIN05	Strategic intelligence has strengthened our organizational capabilities.
SME Performance (SMP)	SMP01	AI capabilities have improved overall business performance.
	SMP02	Entrepreneurial competency significantly enhances our performance metrics.
	SMP03	Strategic intelligence has boosted our organization’s competitiveness.
	SMP04	Our organization’s profitability has increased due to AI adoption.
	SMP05	We have achieved sustainable growth by leveraging AI and strategic intelligence.
